# Effects of Vestibular Rehabilitation with Virtual Reality in Adults with Vestibular Dysfunction: A Systematic Review and Meta-Analysis

**DOI:** 10.1055/s-0046-1819714

**Published:** 2026-05-08

**Authors:** Bianca Simone Zeigelboim, Bianca L. Cavalcante-Leão, Alice Helena de Lima Santos Cardoso, Rosane Sampaio Santos, Helio Afonso Ghizoni Teive, Karinna Verissimo Meira Taveira, Geslaine Janaina Bueno dos Santos, Maria Izabel Rodrigues Severiano, Vanessa Luisa Destro Fidêncio, Cristiano Miranda de Araujo

**Affiliations:** 1 Postgraduate Program in Human Communication Health, Speech Therapy Program, Universidade Tuiuti do Paraná, Curitiba, PR, Brazil; 2 Postgraduate Program in Oral Biology, Faculdade de Odontologia de Ribeirão Preto, Universidade de São Paulo, Ribeirão Preto, SP, Brazil; 3 Postgraduate Program in Internal Medicine and Health Sciences, Department of Internal Medicine, Complexo do Hospital de Clínicas da Universidade Federal do Paraná, Curitiba, PR, Brazil; 4 Associated Postgraduate Program in Speech, Language, and Hearing Sciences, Department of Morphology, Center of Biosciences, Universidade Federal do Rio Grande do Norte, Natal, RN, Brazil

**Keywords:** rehabilitation, vestibular diseases, virtual reality exposure therapy, systematic review

## Abstract

**Introduction:**

Virtual reality is a tool used in the rehabilitation of neurodegenerative diseases and vestibular disorders, enabling immersion in a playful and illusory world.

**Objective:**

To determine the effects of vestibular rehabilitation with virtual reality in adults with vestibular disorders.

**Data Synthesis:**

Appropriate word combinations were selected and tailored specifically to seven electronic databases. Studies that investigated vestibular rehabilitation with virtual reality in individuals > 18-years-old and diagnosed with vestibular dysfunction were included. The risk of bias and the certainty of the evidence was assessed. A random-effects meta-analysis was performed, with a total of 18 articles included. There was an improvement in the level of confidence in balance scores between baseline and postintervention of 11.22 (95% CI = 8.55–13.88; I ^2^ = 18%). An improvement in the disabling effects caused by dizziness was also observed, with a difference between means in relation to the two periods of −22.76 (95% CI = -28.70 to -16.82; I ^2^ = 88%). The certainty of evidence assessment was very low.

**Conclusion:**

Virtual reality therapy in vestibular disorders has shown efficient results, being a useful, low-cost, and motivating tool in the treatment of these disorders.

## Introduction

Body balance depends on the integrity of the vestibular and somatosensory systems, vision, central coordination, and muscle adjustments. Changes to any of these systems can cause dizziness or vertigo. 1 These symptoms are present in more than 10% of the world population and can be triggered by primary or secondary dysfunction of the vestibular system. Dizziness is one of the most common complaints, responsible for more than 8 million medical interventions in the United States of America. 2

Individuals with chronic vestibular dysfunction may be unable to perform several daily activities causing functional deterioration and decrease in quality of life. 3 Vestibular rehabilitation therapy (VRT) has been one of the most indicated interventions to reduce vestibular symptoms and improve body balance in individuals with labyrinthine dysfunction, regardless of age and symptom duration. 4

This therapy aims to modify the postural control system through specific and repetitive physical exercises under different conditions. It has been emphasized for acting physiologically on the vestibular system, being considered a therapeutic resource, due to its proposal to enhance the central mechanisms of neuroplasticity, adaptation, habituation, and replacement, to obtain vestibular compensation. 1

Virtual reality is a tool used in the rehabilitation of neurodegenerative diseases and vestibular disorders, enabling immersion in a playful and illusory world, 5 in which environmental perception is modified by artificial elements, providing a wide variety of stimuli and favoring the rehabilitation process.

Few systematic reviews were performed to assess the effectiveness of the use of virtual reality as a therapeutic resource in vestibular disorders, 6 7 including investigation of its use vestibular rehabilitation of children and adolescents with hearing loss. 8 Still, more detailed searches are needed, involving different databases and adding to the evidence of virtual reality use in VRT.

Thus, this systematic review aimed to answer the following focused question: What are the effects of vestibular rehabilitation with virtual reality in adults with vestibular disorders?

## Review of Literature

This systematic review was reported in accordance with the Preferred Reporting Items for Systematic Reviews and Meta-Analysis (PRISMA) statement. 9

### Eligibility Criteria

The Population, Intervention, Comparison, Outcomes, Studies (PICOS) strategy was used to define the eligibility criteria: Population as adults aged 18 years or older with vestibulopathy of any etiology; Intervention as studies that have used virtual reality as a form of intervention; Comparison (C) as pre- and posttherapy; Outcomes as assessment of vestibular dysfunction using validated questionnaires or tests; and Studies as randomized controlled studies, non-randomized controlled studies, quasi-randomized, and cohort studies;

### Inclusion Criteria

Prospective longitudinal studies investigating vestibular rehabilitation with virtual reality in adults aged 18 years or older with a diagnosis of vestibular dysfunction of any etiology, regardless of gender or ethnicity, were included. Vestibular function must have been assessed using validated tests and questionnaires. All studies that met these criteria were included, regardless of language or time of publication.

### Exclusion Criteria

We excluded studies with individuals under 18 years of age and over 80 years of age; with a sample composed of individuals without a diagnosis of vestibular dysfunction, or where this diagnosis has not been confirmed by a specific test, or where a validated questionnaire has not been used to assess vestibular function, or with patients on any medication associated with therapy; that have not used virtual reality as a form of therapy for vestibular rehabilitation; as well as reviews, letters, books, cross-sectional studies conference abstracts, case reports, case series, opinion articles, technique articles, and guidelines.

### Information Sources and Search Strategy

Search strategies were developed and adapted to seven databases: CINAHL, Cochrane Library, Embase, Latin American Caribbean Literature on Health Sciences (LILACS), PubMed/Medline, Scopus, and Web of Science. An additional partial search of gray literature was performed on Google Scholar, OpenGrey, and ProQuest. All searches in the database were performed on July 24, 2022, with an update on January 17, 2024 ( Supplementary Appendix 1 ). Additionally, a manual search was performed in the references of the articles included, to select those that could have potential for inclusion. All references were managed with the reference manager Endnote X7 (Clarivate Analytics), in which references were stored and duplicate articles were removed. An expert on the subject was also consulted for evaluation regarding the inclusion of any article relevant to the topic.

### Selection Process

Study selection was performed in two phases. In phase 1, two independent authors (AHLS and BLCL) read the titles and abstracts of all selected references, and those studies that met the eligibility criteria were selected for the next phase. In phase 2, the full text was read independently by the same authors as the references selected in the first phase, and again the eligibility criteria were applied. In case of conflict between the two reviewers, when there was no consensus, a third author (BSZ) was involved in making the final decision.

### Data Collection Process and Data Items

For data collection, two authors (AHLS and BLCL) independently collected information from the included studies and compared the information to ensure the integrity of the contents. The data collected consisted of characteristics of the included studies (author, year of publication, country, and study design), sample characteristics (sample size, presence of vestibular dysfunction), main results and conclusion.

When data from the included studies were incomplete or absent, three attempts were made to contact the authors by email to obtain unpublished information. When there was no response, the article was excluded, with due justification.

### Study Risk of Bias Assessment

The risk of bias tool was chosen based on the included study design. For randomized clinical trials, the “Cochrane Collaboration tool for assessing the risk of bias” was used. 10 When the study was classified as quasi-experimental, the ROBINS-I tool was used. 11 For both tools, when there was insufficient detail reported, the risk of bias was judged to be “not clear,” and the authors of the original study were contacted for more information. The methodology of the selected observational studies was evaluated using the risk of bias tool Meta Analysis of Statistics Assessment and Review Instrument (MASTARI). 12 The risk of bias was categorized as “high” when the study had a “yes” score greater than 49%; “moderate” when the study presented between 50 and 69% of a “yes” score; and “low” when the study presented more than 70% of a “yes” score, for the bias risk questions.

The evaluation was performed by two independent reviewers (BLCL and CMA), and when there was no consensus, a third reviewer (KVMT) was involved for the vote.

### Effect Measures

The mean difference between the scores obtained in the pretherapy moment (baseline) and the posttherapy moment were compared by assessing the mean effect of VRT with virtual reality in adults with vestibular disorders.

### Synthesis Methods

The summary effect estimates were calculated as mean differences and synthesized using a random-effects model, weighted by the inverse variance method, with the DerSimonian and Laird estimator used to calculate the variance of the analysis (Tau²). The forest plot was generated using R software (R Foundation for Statistical Computing), version 4.0.2, and all analyses were performed with a significance level of 5%, followed by 95% confidence intervals.

### Reporting Bias Assessment

Publication bias was assessed graphically by analyzing the symmetry of the funnel plot. Additionally, Egger's test, with a significance level of 5%, was applied to detect any potential asymmetries in the funnel plot.

Subgroup analyzes were also performed for estimates composed of at least one study considered as indirect evidence (sample with partial diagnosis of vestibular dysfunction). Furthermore, as in retrospective studies there is no control of the intervention, these were also considered indirect evidence, and analyzes excluding these studies were performed to verify whether there was any change in the estimated effect size and in the degree of heterogeneity.

### Certainty Assessment

The Grading of Recommendations Assessment, Development, and Evaluation (GRADE) system was used to evaluate the quality of evidence. Risk of bias, inconsistency, indirectness, imprecision, and publication bias were evaluated by two authors (KVMT and CMA), and the quality of evidence was classified into four levels: high, moderate, low, and very low. Disagreements were resolved by consensus, and a third reviewer (BLCL) was consulted if necessary.

### Study Selection

**Fig. 1:**
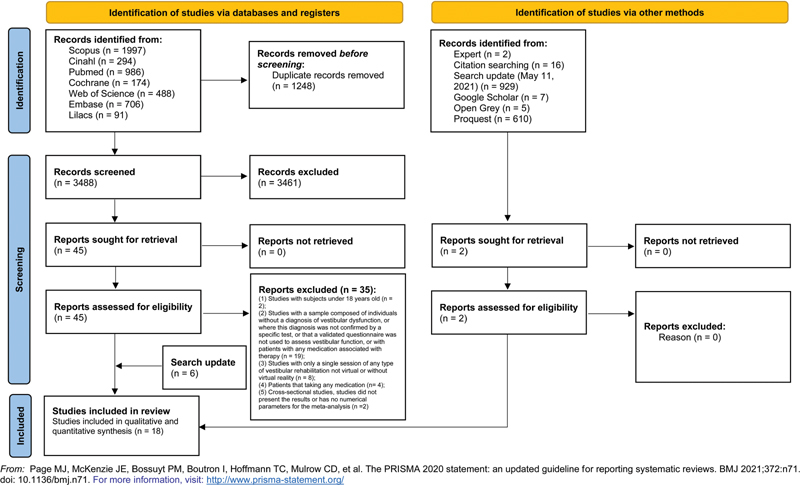
Flowchart of literature search and selection criteria.

The search strategy of all databases resulted in 4,736 studies, leaving 3,488 studies after removal of duplicates. After reading the titles and abstracts (phase 1), 45 studies were selected for reading the full text, and 35 studies were excluded at this stage for not meeting the eligibility criteria ( Supplementary Appendix 2 ). One additional study 13 was included in the analysis, indicated by the experts' suggestion, and one article was included in the search update, thus totaling 18 articles included in the synthesis. Additionally, additional data were obtained from the authors for inclusion in the meta-analysis of two articles. 14 15 As such, six studies were included after the search update ( Fig. 1 ). 16 17 18 19 20 21

Part of the studies met the eligibility criteria, however, only part of the sample was diagnosed with vestibular dysfunction. 13 14 15 22 A retrospective study that met the eligibility criteria was also added to the analysis. These studies were added in the synthesis as a form of indirect evidence; however, they were analyzed differently with appropriate analyses.

### Study Characteristics

Among the included studies, there were 12 clinical trials. Sample sizes ranged from 16 to 87 subjects, and the age range of participants was from 18 to 82 years. Vestibular dysfunction in the samples from the included studies were related to peripheral, central, or mixed vestibular disorders, 2 multiple sclerosis with impaired balance associated with demyelinated lesions in the cerebellum, 23 chronic peripheral vestibular disease, 1 17 unilateral peripheral vestibular hypofunction, 3 23 24 25 peripheral vestibular deficit, 3 16 18 , visual vertigo, 19 subacute stroke, 20 concussion, 21 Parkinson's disease, 15 22 and spinocerebellar ataxia. 13 14 All characteristics of the included studies are available in Table 1 .

**Table 1:** Characteristics of the included studies

Author, Year	Study design	Participants	Intervention	Outcomes measures	Results	Conclusions
Alahmari et al., 2 2014	Clinical Trial	N = 38 Age = 27–78 years Gender = M: 7, F: 31	E: VR 6 sessions (45–60 min, 1 time/week) C: Physiotherapy exercise 6 sessions (45–60 min, 1 time/week)	ABC DHI SCQ DGI FGA TUG SOT VAS SSQ	The results showed a significant improvement in self-report and performance measures in both interventions. There was no difference between groups. The symptoms presented were reduced at the end of treatment.	Although the study did not show a significant difference in the use of the two types of intervention, the mechanism by which patients showed improvement in dizziness and imbalance may be different in both procedures performed.
Başoğlu et al., 16 2022	Quasi-experimental (before and after)	N = 25 Age = 18–65 years Gender = M: 10, F: 15	'Danger Ball', 'Beats Saber', and 'Shooting Rage' balance-based game systems via the “Sony Playstation 4 Virtual Reality” 8 sessions (30–40 min, 2 times/week)	CDP DHI SOT ADT LOS RWS Cybersickness Survey	Patients' DHI scores showed a drastically decreased after intervention and continued to decline in the 8-week follow-up period. All patients' values in SOT, ADT, LOS, and RWS parameters were statistically significant. However, there was no statistically significant difference at 8-week follow-up.	The symptoms of peripheral vestibular hypofunction were reduced, and quality of life improved after VR-based treatment. Therefore, the protocol created has remained effective in these populations in the midterm.
Gutiérrez et al., 26 2013	Clinical Trial	N = 50 Age = 20–60 years Gender = F: 27, M: 20	E: telerehabilitation 40 sessions (20 min, 4 times/week) C: (40 min, 2 times/week) 10 weeks	SOT MCT BBT Tinetti test	Improvement in relation to the general balance in both groups. Visual preference, the contribution of vestibular information, the average response time and the Tinetti test showed significant differences in the experimental group. ANOVA reveals significant differences between groups after treatment in the composite balance score, the Berg and Tinetti scales in the experimental group.	It suggests that the VR program allows for early mechanisms of postural control and response, and can serve as a successful therapeutic alternative in situations where conventional therapy is not readily available.
Hasimova et al., 17 2023	Randomized Clinical Trial	N = 87 Age = 19–70 years Gender = M: 44, F: 43	E: VR in addition to home exercise program. Games with Nintendo Wii device, 16 sessions (45 min, 2 times/week) C: home exercise program (30 minute, 5 times/week)	VAS DHI TUG BBT Romberg	There was no statistical difference between the groups in DHI, TUG, and BBT scores. However, it was determined that the frequency of attacks and daily frequency values were significantly improved in the treatment group compared with the control group	It was determined that the addition of VR therapy increased the effectiveness of rehabilitation
Kanyılmaz et al., 18 2022	Randomized Clinical Trial	N = 26 Age = 65+ years Gender = M: 10, F: 16	E: VR two 3D videos played with a smartphone attached to a VR goggle. Exercises were conducted while sitting and standing (1 ^st^ video), and on the treadmill (2 ^nd^ video). C: performed the exercises in a clinical setting without VR. 15 sessions (35 min, 5 times/week) in both groups	VSS DHI BBT Posturog. TUG IFES GDS HAS	At the end of the 3-week treatment, the effect of exercise using VR on the improvement of the emotional score of DHI was superior to the control group. There were significantly greater improvements in the VSS, all DHI-subgroups and total scores, BBT, HAS between groups at 6-months after the treatment. There were no statistically significant differences between groups in terms of postural stability, fear of falling, and depression at 6-months after the treatment.	VR can be an effective treatment in reducing dizziness, disability due to dizziness, balance, functional mobility, and anxiety in elderly patients with dizziness.
Malisky et al., 13 2024	Quasi-experimental (before and after)	N = 28 Age = 15–70 years Gender = M: 20, F: 8	Vestibular rehabilitation with VR (Wii platform) - 20 sessions (50 min, 2 times/week). Submitted to the same assessment questionnaires before and after the end of the rehabilitation sessions.	VADL ABC	Comparison results between the evaluation time (T1, T2, and T3) with the dimensions (functional, locomotion, and instrumental) of the VADL questionnaire and the results of the ABC questionnaire, showed no difference significant ( *p* > 0.05). The result of the correlation between the questionnaires showed a significant result in all cases. Comparison of the result of vestibular rehabilitation (1 ^st^ to 10 ^th^ session) showed a significant difference in all applied games, except for Ski Slalom. Comparison of vestibular rehabilitation results (1 ^st^ to 20 ^th^ session) showed a significant difference in all applied games.	Direct improvement in quality of life reflected in the reduction in the frequency of falls, in the improvement of balance and gait, thus providing patients with greater self-confidence in carrying out daily life tasks. The importance of rehabilitation with VR was also emphasized due to the improvement of symptoms in this population.
Mandour et al., 19 2022	Clinical Trial	N = 60 Age = 18–65 years	E: VR environment using smartphone-based HMD using videos C: optokinetic stimulation 8 sessions (2 per week)	VVM DHI	There was no statistically significant difference between groups.	Both VR and optokinetic stimulation rehabilitation are equally effective in vestibular rehabilitation in visual vertigo patients supported with home-based exercises.
Manso et al., 1 2016	Clinical Trial	N = 40 Age = 23–63 years Gender = M: 9, F: 31	E: visual/VR stimuli 12 sessions (40 min, 2 times/week) C: conventional rehabilitation, Cawthorne-Cooksey, 12 sessions (40 min, 2 times/week)	DHI EVE Sensitized Romberg test	In the application of DHI, VAS, and the sensitized Romberg test, there was no difference between the experimental and control groups, before and after the intervention ( *p* > 0.005). After the intervention, the experimental and control groups showed lower values ( *p* < 0.05) in DHI and VAS, and higher values ( *p* < 0.05) in the sensitized Romberg test, in some evaluated conditions.	The inclusion of visual stimuli by digital images in the rehabilitation of body balance is effective in reducing dizziness, improving quality of life and postural control in peripheral vestibular disorders.
Meldrum et al., 23 2015	Clinical Trial	N = 71 Control group (mean age: 50.47 years) Study group (mean age: 57.83 years)	E: –VR 30 sessions (15 min, 5 times/week) C: Physiotherapy exercise 30 sessions (15 min, 5 times/week)	VRBQ ABC DGI GS SOT HAD DVA	In both therapies, the groups improved. There was no significant difference in gait speed between groups in the postintervention. There was no significant difference between groups in SOT scores or any other secondary results. In both groups, adherence to the exercises was high, but the group based on VR reported significantly more pleasure, less difficulty and less tiredness in performing the exercises.	Vestibular rehabilitation based on NWFP was not superior to conventional rehabilitation in patients with unilateral vestibular loss in the short or long term, but it may present a more pleasant and less difficult method to perform balance training.
Micarelli et al., 3 2017	Randomized Clinical Trial	N = 47 (with 23 HDM + VR group / 24 VR group) Group 1 (HDM + VR): M: 14, F: 9; aged 49.72 ± 10.34 years Group 2 (VR): M: 13, F: 11; aged 50.48 ± 9.12 years	Both patient HMD unilateral vestibular hypofunction groups underwent vestibular rehabilitation, whereas only the experimental group also underwent the home-based HMD protocol (HMD group).	DHI ABC ZUNG DGI Experimental group: SSQ	The two groups went through a period of 4 weeks rehabilitation protocol simultaneously. A significant effect between posttreatment was found, and the HMD group showed an overall improvement in VOR gain on the lesional side, in posturography parameters, in low frequency spectral domain, as well as in the Italian inventory of dizziness disadvantages and activity-specific balance confidence scale scores. Meanwhile, simulator disease quiz scores demonstrated a significant reduction in related symptoms for experimental home game tasks during HMD procedure. Our results revealed the possible advantages of HMD implementation in vestibular rehabilitation, suggesting it as an innovative, self-evaluated, low-cost, and compatible tool, useful to maximize vestibular rehabilitation results.	This study highlighted those possible advantages related to the HMD implementation in vestibular rehabilitation. In particular, HMD could be proposed as an innovative, ecological, self-assessing, low-cost, and compliant tool, useful in maximizing vestibular rehabilitation outcomes.
Micarelli et al., 24 2019	Clinical Trial	N = 47 (with 23 HDM + VR group / 24 VR group) Group 1: 12 elderly; mean age 74.3 ± 4.7 years; M: 6, F: 6. 12 people with MCI, mean age 72.5 ± 3.6, M: 5, F: 7 Group 2: 11 elderly people, mean age 76.9 ± 4.7; M: 5, F: 6. 12 elderly people with MCI, mean: 76.3 ± 5.5; M: 5, F: 7 Group 1 and 2 UVH	The quality-of-life scores were collected from 12 elderly people with UVH and 12 UVH individuals who suffer from MCI only submitted to vestibular rehabilitation; as well as in 11 elderly people with UVH and 12 UVH individuals suffering from MCI undergoing a home-based HMD + vestibular rehabilitation protocol.		Although the intrasubject analysis in all groups found a significant improvement ( *p* < 0.05) in posturographic parameters and scores related to dizziness and quality of life, with no change in VOR gain, the implementation of HMD showed a significant increase ( *p* < 0.05) in comparisons between groups after treatment in the same tests, as well as VOR gain in relation to older adults and participants with only MCI in vestibular rehabilitation.	This study demonstrates that the implementation of a VR protocol at home can be a safe option to improve vestibular dysfunction, postural control, and quality of life in vestibular impairment in patients when cognitive decline could hinder the achievement of the goal.
Pavlou et al., 4 2012	Clinical Trial	N = 16 Age = 18–75 years Gender = M: 9, F: 7	E: dynamic VR 8 sessions (45 min, 2 times/week) C: static VR 8 sessions (45 min, 2 times/week)	SVQ BDI BAI FQ DGI SCQ BDS BAS FQ DGI	Significant differences were observed between Groups D (performed dynamic VR exercises) compared with Group S (performed static VR exercises) for symptoms of visual vertigo, with the first two showing a significant improvement of 59, 2, and 25.8%, respectively. Depression scores improved only for Group S ( *p* = 0.01), while a trend of significance was observed for Group D in relation to anxiety scores ( *p* = 0.07).	Exposure to dynamic VR environments should be considered as a useful complement to vestibular rehabilitation programs in patients with peripheral vestibular disorders.
Sana et al., 20 2023	Randomized Clinical Trial	N = 30 Age = 40–70 years Gender = M: 13, F: 17	E: VR training Wii console, Wii Balance Board, Nintendo Wii remote control, and Wii Fit Plus software. C: exercises to improve gaze stability, balance, gait, and somatosensory integration. 24 sessions (20 min, 3 times/week, for 8 weeks)	TUG DGI DHI	A significant improvement was observed in the physical, emotional, and functional domains of DHI between both groups. VR showed significant improvements in the DGI compared with the vestibular group.	VR has a greater effect on improving balance and gait in people with subacute stroke. Vestibular rehabilitation without VR has a greater impact on reducing dizziness.
Santos et al., 14 2017	Quasi-experimental (before and after)	N = 28 Age = 15–70 years Gender = M: 20, F: 8	Vestibular rehabilitation with VR (Wii platform) 20 sessions (50 min, 2 times/week). Submitted to the same assessment questionnaires before and after the end of the rehabilitation sessions.	DHI BBS SF-36	Regarding to baseline, the participants showed significant improvements posttraining in their DHI and BBS scores. These improvements were accompanied by improvements in perceived quality of life as measured by the SF-36. There were significant improvements in balance and gait, fall frequency, and patients' self-confidence.	Showed consistent improvement postrehabilitation in their ability to play VR games (i.e., Soccer Heading, Tightrope, Table Tilt, and Ski Slalom). VR rehabilitation may be effective as a therapy for spinocerebellar ataxia. The breadth of improvements evidenced here should promote physical and psychological recovery, while fostering a better quality of life.
Sessoms et al., 21 2023	Randomized Clinical Trial	N = 38 Age = 18–55 years	E1: VR Computer Assisted Rehabilitation Environment (CAREN Extended, Motekforce Link BV) E2: hybrid VR and conventional vestibular therapy C: conventional vestibular physical therapy 12 sessions (30 min, 2 times/week, for 6 weeks)	ABC DHI SOT FGA	Conventional and hybrid therapy groups demonstrated significant improvements in patient reported confidence and function earlier in the treatment course. In VR-based therapy this did not happen. In the comparison of pre-to-post-effects, there was no clear superiority or inferiority observed in either of the experimental treatments observed.	Conventional vestibular therapy, VR-based vestibular therapy or a combination of both interventions results in improved patient reported, clinician-assessed, and instrumented measures of balance and function following 6 weeks of treatment.
Severiano et al., 14 2018	Quasi-experimental (before and after)	N = 16 Age = 18–82 years Gender = M: 10, F: 6	Vestibular rehabilitation with VR (Wii platform) - 20 sessions (50 minute, 2 sessions per week). Submitted to the same assessment questionnaires before and after the end of the rehabilitation sessions.	DHI BBS SF-36 SRT	The final scores on the DHI and the BBC were better after rehabilitation. The SRT showed a significant result after rehabilitation. The SF-36 showed a significant change in the functional capacity for the Tightrope Walk and Ski Slalom VR games ( *p* < 0.05), as well as in the mental health aspect of the Ski Slalom game ( *p* < 0.05). DHI and BBC showed significant changes in the Ski Slalom game ( *p* < 0.05). There was evidence of clinical improvement in patients at the final assessment after virtual rehabilitation.	The rehabilitation of the body balance by means of VR proved efficient in improving body balance and functional capacity, reducing fall risk, increasing self-confidence, and enhancing the quality of life of patients suffering from PD. The Tightrope Walk and Ski Slalom virtual games were shown to be the most effective for this population.
Verdecchia et al., 25 2017	Retrospective Cohort	N = 69 Median age = 64-years-old Gender = M: 28, F: 41	Patient records were reviewed between April 2009 and May 2011 from the vestibular rehabilitation area of a university hospital. The variables studied were the DHI, DGI, and DVA. All subjects used Wii as a complement.	DHI IDM DVA	The initial median DHI score was 40 points (range 0–84, 25–75%: 20–59) and the final, 24 points (range 0–76, 25–75% = 10.40), *p* < 0.0001. The initial median for the DGI score was 21 points (range 8–24, 25–75%: 17.5–2.3) and the final, 23 (range 12–24, 25–75%: 21–23), *p* < 0.0001. The initial median for DVA was 2 (range 0–6, 25–75%: 1–4) and the final, 1 (range 0–3, 25–75%: 0–2), *p* < 0.0001.	A reduction was observed in the DHI values. DGI values increased and DVA improved. All these variations were statistically significant.
Zeigelboim et al., 22 2021	Quasi-experimental (before and after)	N = 16 Age = 18–82 years Gender = M: 10, F: 6	Vestibular rehabilitation with VR (Wii platform): 20 sessions (50 min, 2 times/week). Patients who completed the same assessment questionnaires before and after rehabilitation were instructed not to perform any type of complementary or traditional therapies.	VADL ABC	ABC was statistically significant between the first and second assessments. The correlation between the questionnaires showed a statistically significant result in the walking subscale, and the functional subscale was only verified in the second evaluation. Patients showed clinical improvement in the final assessment after rehabilitation with significant result for Tightrope Walking and Slalom Ski games.	Results showed that the VADL and ABC questionnaires, applied before and after rehabilitation, were important tools to measure patients' independence, confidence, and balance while developing daily activities. The questionnaires may effectively contribute to quantify the effect of applied therapeutics and, consequently, their impact on the quality of life of PD patients.

**Abbreviations:** ABC, Activities-Specific Balance Confidence Scale; ADT, Adaptation Test; BAI, Beck Anxiety Scale; ANOVA, analysis of variance; BBS, Berg Balance Scale; BBT, Berg Balance Test; BDI, Beck Depression Scale; C, Control; DGI, Dynamic Gait Index; DHI, Dizziness Handicap Inventory; DVA, Dynamic Visual Acuity; E, Experimental; F, Female; FGA, Functional Gait Assessment; FQ, Fear Questionnaire; GDS, Geriatric Depression Scale; HAS, Hamilton Anxiety Scale; HMD, head-mounted display; IFES, International Falls Efficacy Scale; LOS, Limits of Stability; M, Male; MCI, mild cognitive impairment; Posturograp, Dynamic Posturography Biodex Balance System; RWS, Rhythmic Weight Shift; SCQ, Situational Characteristics Questtionaire; SF-36, 36-Item Short Form Health Survey; SIS, Stroke impact scale; SOT, Sensory Organization Test; SRT, Sitting–Rising Test; SSQ, Simulator Sickness Questionnaire; ST, Stepping Test; TUG, Timed Up and Go; UVH, unilateral vestibular hypofunction; VADL, Vestibular Disorders Activities of Daily Living Scale; VAS, Visual Analogic Scale; VOR, vestibuloocular reflex; VR, virtual reality; VRBQ, Vestibular Rehabilitation Benefits Questionnaire; VSS, Vertigo Symptom Scale; VVM, Modified Arabian Version of Mallinson Questionnaire.

### Risk of Bias in Studies

**Fig. 2:**
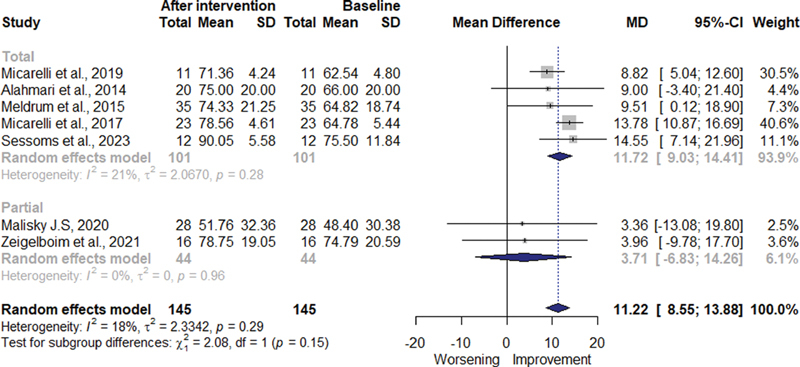
Forest plot of the meta-analysis of the Activities-specific Balance Confidence (ABC) Scale, displaying risk-of-bias judgements for each study included.

The domains in which most studies failed regarding description, bringing incomplete details, were related to selection bias (allocation concealment) and reporting bias. The assessment of bias risk for all included studies can be visualized on Fig. 2 .

### Results of Individual Studies

The most used questionnaires and tests to assess vestibular function were the Activities-specific Balance Confidence (ABC) Scale, Dizziness Handicap Inventory (DHI), Dynamic Gait Index (DGI), and Sensory Organization Test (SOT). The number of sessions ranged from 6 1 to 40. 26 Despite the differences, the studies showed, with the use of VRT, improvement in dizziness and imbalance, improved postural control and response, improved quality of life and postural control in peripheral vestibular disorders, increased values of the DGI, and improved dynamic visual acuity (DVA).

There was disagreement in the literature when analyzing the individual results of each study included in the synthesis. Although all showed significant posttherapy improvement in at least one of the evaluated domains, there was disagreement when considering specific protocols, with studies showing significant improvements ( *p* < 0.05) in the assessments for the ABC 3 24 and DHI, 1 3 18 24 25 and for the DGI 20 23 24 and SOT 16 23 tests. On the other hand, some studies did not demonstrate statistical significance when considering these same assessments. 2 4 13 14 15 17 22 26

### Results of Syntheses

The meta-analysis was performed on the 18 selected studies, which used the ABC, DHI, DGI, and SOT protocols. For all protocols used, a comparison was made between the initial period (baseline) and the postintervention period, with an improvement in the outcomes assessed in two of the four protocols used in the initial analysis.

**Fig. 3:**
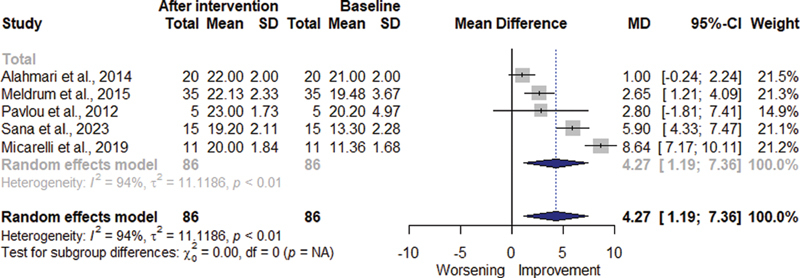
Forest plot of the meta-analysis of the Dynamic Gait Index (DGI), displaying risk-of-bias judgements for each study included.

The ABC questionnaire demonstrated a significant improvement in the level of confidence in balance during a set of daily activities, associated with a wide spectrum of difficulties, with a mean difference in scores between baseline and postintervention of 11.22 (95% CI = 8.55–13.88; I ^2^ = 18%), with higher posttherapy confidence scores with virtual reality. The two studies considered as indirect evidence decreased the effect estimate, and for this reason they were analyzed in different subgroups, showing an average improvement of 11.72 in the postintervention scores not considering these studies (95% CI = 9.03–14.41; I ^2^ = 21%). However, even with the inclusion of indirect evidence, the effect's direction remained the same, denoting an improvement in the confidence in balance during daily activities ( Fig. 2 ). When considering the DGI test, there was also a statistically significant mean difference of 4.27 (95% CI = 1.19–7.36; I2 = 96%), as shown in Fig. 3 .

**Fig. 4:**
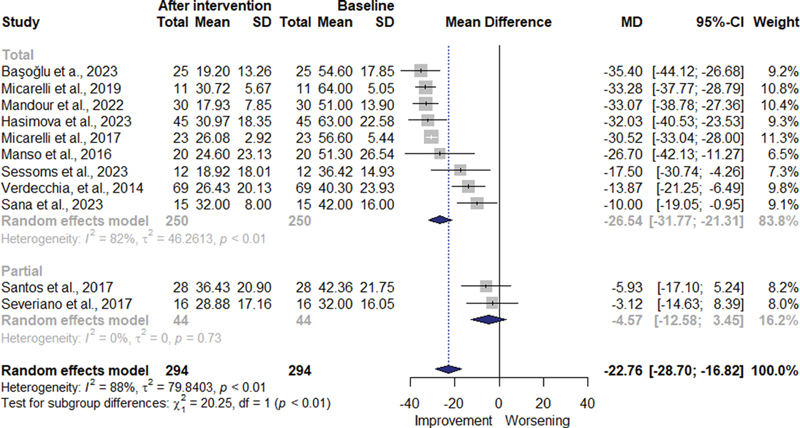
Forest plot of the meta-analysis of the Dizziness Handicap Inventory (DHI), displaying risk-of-bias judgements for each study included.

Among all the studies, the analysis that showed the greatest global effect was when comparing the self-perception of the disabling effects caused by dizziness between the period before and after virtual reality-based vestibular therapy, through the DHI, showing a difference between means in relation to the two periods of −22.76 (95% CI = − 28.70–−16.82; I ^2^ = 88%), as shown in ( Fig. 4 ).

**Fig. 5:**
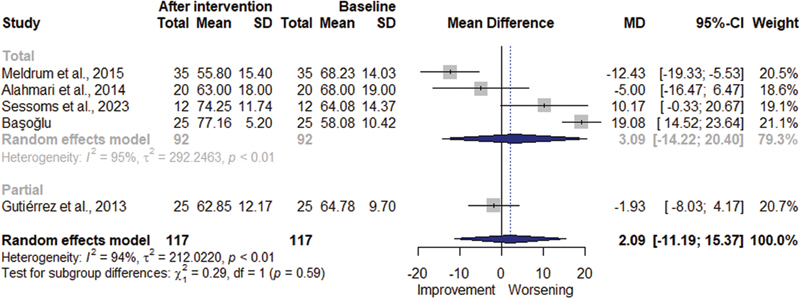
Forest plot of the meta-analysis of the Sensory Organization Test (SOT), displaying risk-of-bias judgements for each study included.

The SOT test did not show statistical significance when comparing pre- and post-therapy, with a mean effect of −2.09 (CI95% = − 11.19–15.37, I ^2^ = 94%), as shown in ( Fig. 5 ).

### Reporting Biases

No publication bias was identified through the graphical analysis of the funnel plot or by Egger's test ( *p* > 0.05).

### Certainty of Evidence

The certainty of evidence identified using GRADE 27 was considered very low. The main reasons were a lack of information in several domains, making correct judgment impossible. The inconsistency was also considered serious in meta-analyses with I ^2^ values > 60%, suggesting a very low confidence in the estimated effect. The difference between the study populations, since information about the etiology of vestibular disorders is different between the groups. Finally, the inaccuracy related to the small sample size and number of events were also considered serious ( Table 2 ).

**Table 2:** Summary of findings

Certainty assessment						
Participants	Risk of bias	Inconsistency	Indirectness	Imprecision	Publication bias	Overall certainty of evidence
Studies						
Follow-up (Varied across studies - from immediate post-intervention to 6 months)						
**ABC**						
(3 RCTs; n = 145)	serious ^a^	not serious	serious ^b^	serious ^c^	none	
						VERY LOW
**DGI**						
(4 RCTs; n = 86)	serious ^a^	serious ^d^	serious ^b^	serious ^c^	none	
						VERY LOW
**SOT**						
(3 RCTs; n = 117)	serious ^e^	not serious ^f^	serious ^b^	serious ^c^	none	
						VERY LOW
**DHI**						
(2 RCTs; n = 294)	serious ^a^	not serious	serious ^b^	serious ^c^	none	
						VERY LOW

**Abbreviations:** ABC, Activities-Specific Balance Confidence Scale; CI, confidence interval; DGI, Dynamic Gait Index; DHI, Dizziness Handicap Inventory; RCT, randomized controlled trial; SOT, Sensory Organization Test.

**Notes:**^a^ Problems with randomization and allocation concealment. ^b^ The etiology of vestibular disorders is different between groups. ^c^ The sample size or the number of events does not meet the optimal information (Cochrane handbook, Chapter 14). ^d^ Serious inconsistency: large heterogeneity I2 = 64%, *p* -value = 0.04, and variability in CIs. ^e^ Problems with allocation concealment and selective outcome report. ^f^ Serious inconsistency: large heterogeneity I ^2^ = 60%, *p* -value = 0.08, and variability in CIs.

## Discussion

This systematic review and meta-analysis investigated the effects of VRT with virtual reality in adults with vestibulopathy of any etiology. Based on the literature, this treatment can be a successful way to facilitate the desensitization of symptoms resulting from sensory conflict between the visual, vestibular, and somatosensory systems. 2 Another advantage of virtual reality rehabilitation is to provide a safe environment for patients at risk of falling, hold their attention for a longer period and immediate feedback making therapy more exciting, increasing their motivation. 28

Imbalance places individuals at a higher risk of falls, making it difficult to perform daily activities. 29 Verdecchia et al. 25 observed a reduction in the perception of disability, an increase in DGI scores, and an improvement in gaze stability following treatment with virtual rehabilitation. Alahmari et al. 2 found statistically significant improvements in the ABC, DHI, DGI, and SOT outcome measures. According to Micarelli et al., 24 interventions resulted in significant outcomes in the DGI and ABC scores across all study groups. These data partially corroborate the findings of the present study, where the ABC questionnaire showed an improvement in the level of confidence in balance across a set of daily activities. However, it is not possible to assert with high certainty, based on the current literature, that there is a reduced risk of falls when considering the postvirtual therapy DGI test. Confounding factors, such as age, may have influenced the effect estimate, increasing the uncertainty of this result. Sana et al. 20 evaluated subacute stroke patients aged between 40 and 70 years and observed that virtual reality-based treatment was more effective than conventional VRT in improving balance and gait, with significant improvement in DGI scores.

In this systematic review, the largest effect size found by the meta-analytic analysis was when considering the DHI questionnaire, which validates the individual's perception of their activities of daily living. As the rehabilitation takes place, there is an improvement in proprioception, the ability to recognize the spatial location of his body, its position and orientation. Consequently, they can better perceive the differences in the performance of daily activities.

For Sasaki et al., 30 vestibular dysfunction can cause gait change, with slow walking, lateral deviations, wider support base, and restrictions during voluntary rotation of the trunk and head, due to the sensation of dizziness and postural instability. In this sense, Santos et al. 14 observed a significant improvement in the DHI in 28 patients diagnosed with spinocerebellar ataxia who used VRT with virtual reality, in the pre- and postintervention scores, reporting that the participants experienced a relief from the harmful effects from dizziness.

The SOT test is performed by computerized dynamic posturography, the symmetry of patients' weight is shown before and after the force platform translations, indicating whether the patient maintains uniform weight during the procedure. Meldrum et al. 23 evaluated VRT with virtual reality using Wii Fit Plus (Nintendo Co., Ltd.) for balance exercises, however, the authors observed that the results found were not superior to conventional VRT. Gait speed improved in both groups with a magnitude of improvement that is in agreement with other studies using conventional VRT. However, the use of the SOT was not completely adequate, as no superior effects were found in the scores, with both groups improving on average 12%, but without statistical significance.

Sessoms et al. 21 evaluated 38 active-duty United States military service members with persistent balance impairment resulting from concussion and observed that the SOT significantly improved early in treatment in conventional VRT and virtual reality-based treatment. Likewise, in this review, no statistically significant differences were found when considering this test, including studies with direct and indirect evidence. On the other hand, when only the studies with direct evidence were kept, the analysis showed significance, also decreasing the level of heterogeneity that exists. More studies that provide direct evidence for this outcome should be performed to increase the certainty of this evidence.

Some limitations should be pointed out, such as the uncertainty in some outcomes, and the existing methodological differences, such as the number of sessions and the etiology of vestibular dysfunction. On the other hand, the analyzes performed provided a decrease in heterogeneity in all assessments. Thus, this synthesis points to the effectiveness of virtual reality in improving vestibular dysfunction in different domains. In individuals undergoing virtual therapy, however, the evidence currently available still only provides a low certainty of evidence, requiring studies with a better description methodological approach to ensure greater robustness to these findings.

## Final Comments

Based on this meta-analysis, it was observed that VRT with virtual reality showed improved balance confidence, lower risk of falling and self-perception of the disabling effects caused by dizziness, and improved postural stability. Thus, it is concluded that virtual reality therapy in vestibular disorders has shown efficient results, being a useful, low-cost, and motivating tool in their treatment.
