# Supplementary material for: Effects of Vestibular Rehabilitation with Virtual Reality in Adults with Vestibular Dysfunction: A Systematic Review and Meta-Analysis
Source: Int Arch Otorhinolaryngol. 2026 May 8;30(2):1–14. doi: 10.1055/s-0046-1819714 (PMC13288422; doi:10.1055/s-0046-1819714)
Supplement: Supplementary file 1 — Supplementary Material [file 10-1055-s-0046-1819714-s241858.pdf]

Supplementary Appendix 1 Search strategy used in electronic databases

Database	Search
**Cinahl**	(“Balance Therapy” OR “Desensitization” OR “Vestibular Physical Therapy” OR “Vestibular Rehabilitation” OR “Virtual Games” OR “Virtual Reality Exposure Therapy” OR “Virtual Reality Immersion Therapy” OR “Virtual Reality Therapies” OR “Virtual Reality Therapy”) AND (“Bilateral Vestibular Deficiency” OR “bilateral vestibulopathy” OR “Bilateral Vestibular Loss” OR “Conjugate Nystagmus” OR “Convergence Nystagmus” OR “Dissociated Nystagmus” OR “dizziness” OR “Fatigable Positional Nystagmus” OR “Horizontal Nystagmus” OR “Jerk Nystagmus” OR “Multidirectional Nystagmus” OR “pathologic nystagmus” OR “Pendular Nystagmus” OR “Periodic Alternating Nystagmus” OR “Permanent Nystagmus” OR “Rebound Nystagmus” OR “Retraction Nystagmus” OR “Rotary Nystagmus” OR “Rotational Nystagmus” OR “See-Saw Nystagmus” OR “Spontaneous Ocular Nystagmus” OR “Symptomatic Nystagmus” OR “Temporary Nystagmus” OR “Unidirectional Nystagmus” OR “Vertical Nystagmus” OR “Vestibular Areflexia” OR “Vestibular Disease” OR “Vestibular Diseases”)
**PubMed**	1. (“Balance Therapy”[All Fields] OR “Desensitization”[All Fields] OR “Vestibular Physical Therapy”[All Fields] OR “Vestibular Rehabilitation”[All Fields] OR “Virtual Games”[All Fields] OR “Virtual Reality Exposure Therapy”[All Fields] OR “Virtual Reality Exposure Therapy”[MeSH Terms] OR “Virtual Reality Immersion Therapy”[All Fields] OR “Virtual Reality Therapies”[All Fields] OR “Virtual Reality Therapy”[All Fields])<br>2. (“Bilateral Vestibular Deficiency”[All Fields] OR “bilateral vestibulopathy”[MeSH Terms] OR “bilateral vestibulopathy”[All Fields] OR “Bilateral Vestibular Loss”[All Fields] OR “Conjugate Nystagmus”[All Fields] OR “Convergence Nystagmus”[All Fields] OR “Dissociated Nystagmus”[All Fields] OR “dizziness”[All Fields] OR “Fatigable Positional Nystagmus”[All Fields] OR “Horizontal Nystagmus”[All Fields] OR “Jerk Nystagmus”[All Fields] OR “Multidirectional Nystagmus”[All Fields] OR (“nystagmus, pathologic”[MeSH Terms] OR “pathologic nystagmus”[All Fields] OR “Nystagmus, Pathologic”[All Fields] OR “Pathologic Nystagmus”[All Fields] OR “Pendular Nystagmus”[All Fields] OR “Periodic Alternating Nystagmus”[All Fields] OR “Permanent Nystagmus”[All Fields] OR “Rebound Nystagmus”[All Fields] OR “Retraction Nystagmus”[All Fields] OR “Rotary Nystagmus”[All Fields] OR “Rotational Nystagmus”[All Fields] OR “See-Saw Nystagmus”[All Fields] OR “Spontaneous Ocular Nystagmus”[All Fields] OR “Symptomatic Nystagmus”[All Fields] OR “Temporary Nystagmus”[All Fields] OR “Unidirectional Nystagmus”[All Fields] OR “Vertical Nystagmus”[All Fields] OR “Vestibular Areflexia”[All Fields] OR “Vestibular Disease”[All Fields] OR “Vestibular Diseases”[All Fields])<br>3. #1 AND #2
**SCOPUS**	TITLE-ABS-KEY(“Balance Therapy” OR “Desensitization” OR “Vestibular Physical Therapy” OR “Vestibular Rehabilitation” OR “Virtual Games” OR “Virtual Reality Exposure Therapy” OR “Virtual Reality Immersion Therapy” OR “Virtual Reality Therapies” OR “Virtual Reality Therapy”) AND TITLE-ABS-KEY(“Bilateral Vestibular Deficiency” OR “bilateral vestibulopathy” OR “Bilateral Vestibular Loss” OR “Conjugate Nystagmus” OR “Convergence Nystagmus” OR “Dissociated Nystagmus” OR “dizziness” OR “Fatigable Positional Nystagmus” OR “Horizontal Nystagmus” OR “Jerk Nystagmus” OR “Multidirectional Nystagmus” OR “pathologic nystagmus” OR “Pendular Nystagmus” OR “Periodic Alternating Nystagmus” OR “Permanent Nystagmus” OR “Rebound Nystagmus” OR “Retraction Nystagmus” OR “Rotary Nystagmus” OR “Rotational Nystagmus” OR “See-Saw Nystagmus” OR “Spontaneous Ocular Nystagmus” OR “Symptomatic Nystagmus” OR “Temporary Nystagmus” OR “Unidirectional Nystagmus” OR “Vertical Nystagmus” OR “Vestibular Areflexia” OR “Vestibular Disease” OR “Vestibular Diseases”)
**Web of Science**	1. TS = (“Balance Therapy” OR “Desensitization” OR “Vestibular Physical Therapy” OR “Vestibular Rehabilitation” OR “Virtual Games” OR “Virtual Reality Exposure Therapy” OR “Virtual Reality Immersion Therapy” OR “Virtual Reality Therapies” OR “Virtual Reality Therapy”)<br>2. TS = (“Bilateral Vestibular Deficiency” OR “bilateral vestibulopathy” OR “Bilateral Vestibular Loss” OR “Conjugate Nystagmus” OR “Convergence Nystagmus” OR “Dissociated Nystagmus” OR “dizziness” OR “Fatigable Positional Nystagmus” OR “Horizontal Nystagmus” OR “Jerk Nystagmus” OR “Multidirectional Nystagmus” OR “pathologic nystagmus” OR “Pendular Nystagmus” OR “Periodic Alternating Nystagmus” OR “Permanent Nystagmus” OR “Rebound Nystagmus” OR “Retraction Nystagmus” OR “Rotary Nystagmus” OR “Rotational Nystagmus” OR “See-Saw Nystagmus” OR “Spontaneous Ocular Nystagmus” OR “Symptomatic Nystagmus” OR “Temporary Nystagmus” OR “Unidirectional Nystagmus” OR “Vertical Nystagmus” OR “Vestibular Areflexia” OR “Vestibular Disease” OR “Vestibular Diseases”)<br>**3. #1 AND #2**
**Cochrane Library**	(“Balance Therapy” OR “Desensitization” OR “Vestibular Physical Therapy” OR “Vestibular Rehabilitation” OR “Virtual Games” OR “Virtual Reality Exposure Therapy” OR “Virtual Reality Immersion Therapy” OR “Virtual Reality Therapies” OR “Virtual Reality Therapy”) AND (“Bilateral Vestibular Deficiency” OR “bilateral vestibulopathy” OR “Bilateral Vestibular Loss” OR “Conjugate Nystagmus” OR “Convergence Nystagmus” OR “Dissociated Nystagmus” OR “dizziness” OR “Fatigable Positional Nystagmus” OR “Horizontal Nystagmus” OR “Jerk Nystagmus” OR “Multidirectional Nystagmus” OR “pathologic nystagmus” OR “Pendular Nystagmus” OR “Periodic Alternating Nystagmus” OR “Permanent Nystagmus” OR “Rebound Nystagmus” OR “Retraction Nystagmus” OR “Rotary Nystagmus” OR “Rotational Nystagmus” OR “See-Saw Nystagmus” OR “Spontaneous Ocular Nystagmus” OR “Symptomatic

(*Continued*)

 © 2026. The Author(s).

Supplementary Appendix 1 (*Continued*)

Database	Search
	Nystagmus" OR "Temporary Nystagmus" OR "Unidirectional Nystagmus" OR "Vertical Nystagmus" OR "Vestibular Areflexia" OR "Vestibular Disease" OR "Vestibular Diseases")
**LILACS**	("Terapia de equilíbrio" OR "Desensibilização" OR "Fisioterapia vestibular" OR "Reabilitação vestibular" OR "Jogos virtuais" OR "Terapia de exposição à realidade virtual" OR "Terapia de imersão em realidade virtual" OR "Terapias de realidade virtual" OR "Terapia de realidade virtual" OR "Terapia de equilibrio" OR "Desensibilización" OR "Terapia física vestibular" OR "Rehabilitación vestibular" OR "Juegos virtuales" OR "Terapia de exposición de realidad virtual" OR "Terapia de inmersión en realidad virtual" OR "Terapias de realidad virtual" OR "Terapia de realidad virtual" OR "Balance Therapy" OR "Desensitization" OR "Vestibular Physical Therapy" OR "Vestibular Rehabilitation" OR "Virtual Games" OR "Virtual Reality Exposure Therapy" OR "Virtual Reality Immersion Therapy" OR "Virtual Reality Therapies" OR "Virtual Reality Therapy") AND ("Deficiência vestibular bilateral" OR "vestibulopatia bilateral" OR "Perda vestibular bilateral" OR "Nistagmo conjugado" OR "Nistagmo de convergência" OR "Nistagmo dissociado" OR "tontura" OR "Nistagmo posicional fatigável" OR "Nistagmo horizontal" OR "Nistagmo multidirecional" OR "nistagmo patológico" OR "Nistagmo pendular" OR "Nistagmo alternado periódico" OR "Nistagmo permanente" OR "Nistagmo de rebote" OR "Nistagmo de retração" OR "Nistagmo rotativo" OR "Nistagmo rotacional" OR "Nistagmo giratório" OR "Nistagmo ocular espontâneo" OR "Nistagmo sintomático" OR "Nistagmo temporário" OR "Nistagmo unidirecional" OR "Nistagmo vertical" OR "Areflexia vestibular" OR "Doença vestibular" OR "Doenças vestibulares" OR "Deficiencia vestibular bilateral" OR "vestibulopatía bilateral" OR "Pérdida vestibular bilateral" OR "Nistagmo conjugado" OR "Nistagmo de convergencia" OR "Nistagmo disociado" OR "Nistagmo posicional fatigable" OR "Nistagmo horizontal" OR "Nistagmo multidireccional" OR "nistagmo patológico" OR "Nistagmo pendular" OR "Nistagmo alterno periódico" OR "Nistagmo permanente" OR "Nistagmo de rebote" OR "Nistagmo de retracción" OR "Nistagmo rotatorio" OR "Nistagmo rotacional" OR "Nistagmo en sierra" OR "Nistagmo ocular espontáneo" OR "Nistagmo sintomático" OR "Nistagmo temporal" OR "Nistagmo unidireccional" OR "Nistagmo vertical" OR "Areflexia vestibular" OR "Enfermedad vestibular" OR "Enfermedades vestibulares")
**EMBASE**	('balance therapy':ti,ab,kw OR 'desensitization':ti,ab,kw OR 'vestibular physical therapy':ti,ab,kw OR 'vestibular rehabilitation':ti,ab,kw OR 'virtual games':ti,ab,kw OR 'virtual reality exposure therapy':ti,ab,kw OR 'virtual reality immersion therapy':ti,ab,kw OR 'virtual reality therapies':ti,ab,kw OR 'virtual reality therapy':ti,ab,kw) AND ('bilateral vestibular deficiency':ti,ab,kw OR 'bilateral vestibulopathy':ti,ab,kw OR 'bilateral vestibular loss':ti,ab,kw OR 'conjugate nystagmus':ti,ab,kw OR 'convergence nystagmus':ti,ab,kw OR 'dissociated nystagmus':ti,ab,kw OR 'dizziness':ti,ab,kw OR 'fatigable positional nystagmus':ti,ab,kw OR 'horizontal nystagmus':ti,ab,kw OR 'jerk nystagmus':ti,ab,kw OR 'multidirectional nystagmus':ti,ab,kw OR 'pathologic nystagmus':ti,ab,kw OR 'pendular nystagmus':ti,ab,kw OR 'periodic alternating nystagmus':ti,ab,kw OR 'permanent nystagmus':ti,ab,kw OR 'rebound nystagmus':ti,ab,kw OR 'retraction nystagmus':ti,ab,kw OR 'rotary nystagmus':ti,ab,kw OR 'rotational nystagmus':ti,ab,kw OR 'see-saw nystagmus':ti,ab,kw OR 'spontaneous ocular nystagmus':ti,ab,kw OR 'symptomatic nystagmus':ti,ab,kw OR 'temporary nystagmus':ti,ab,kw OR 'unidirectional nystagmus':ti,ab,kw OR 'vertical nystagmus':ti,ab,kw OR 'vestibular areflexia':ti,ab,kw OR 'vestibular disease':ti,ab,kw OR 'vestibular diseases':ti,ab,kw)
**Google Scholar**	"Virtual Reality Therapy" AND "Vestibular disease"
**Open Gray**	"Virtual Reality Therapy"
**ProQuest**	("Balance Therapy" OR "Desensitization" OR "Vestibular Physical Therapy" OR "Vestibular Rehabilitation" OR "Virtual Games" OR "Virtual Reality Exposure Therapy" OR "Virtual Reality Immersion Therapy" OR "Virtual Reality Therapies" OR "Virtual Reality Therapy") AND ("Bilateral Vestibular Deficiency" OR "bilateral vestibulopathy" OR "Bilateral Vestibular Loss" OR "Conjugate Nystagmus" OR "Convergence Nystagmus" OR "Dissociated Nystagmus" OR "dizziness" OR "Fatigable Positional Nystagmus" OR "Horizontal Nystagmus" OR "Jerk Nystagmus" OR "Multidirectional Nystagmus" OR "pathologic nystagmus" OR "Pendular Nystagmus" OR "Periodic Alternating Nystagmus" OR "Permanent Nystagmus" OR "Rebound Nystagmus" OR "Retraction Nystagmus" OR "Rotary Nystagmus" OR "Rotational Nystagmus" OR "See-Saw Nystagmus" OR "Spontaneous Ocular Nystagmus" OR "Symptomatic Nystagmus" OR "Temporary Nystagmus" OR "Unidirectional Nystagmus" OR "Vertical Nystagmus" OR "Vestibular Areflexia" OR "Vestibular Disease" OR "Vestibular Diseases")

 © 2026. The Author(s).

Supplementary Appendix 2 Excluded articles and reasons for exclusion

Author, Year	Reason for exclusion
Alves et al., 2018[1]	2
Ansai et al., 2016[2]	1
Bayat et al., 2012[3]	3
Bayat et al., 2017[4]	3
Bittar et al., 1999[5]	3
Bittar et al., 2007[6]	3
Bittar et al., 2011[7]	3
Black et al., 2000[8]	3
Ferraz et al., 2018[9]	2
Gandolfi et al., 2017[10]	2
Garcia et al., 2013[11]	4
Kim et al 2009[12]	5
Lee et al., 2015[13]	2
Liao et al., 2015[14]	2
Lin Zhi-Cheng et al., 2016[15]	2
Marioni et al., 2013[16]	4
Marioni et al., 2013[17]	4
Meldrum et al 2012[18]	5
McConville et al., 2012[19]	2
Morozetti et al., 2011[20]	3
Nishiike et al., 2013[21]	2
Ozgonenel et al., 2016[22]	2
Park et al., 2019[23]	2
Pedreira et al., 2013[24]	2
Pompeu et al., 2012[25]	2
Purvis et al., 2015[26]	3
Ribas et al., 2017[27]	2
Shen et al., 2014[28]	2
Shin et al., 2016[29]	2
van den Heuvel et al., 2014[30]	2
Salisbury et al., 2018[31]	2
Sparrer et al., 2013[32]	4
Suarez et al., 2006[33]	1
Yang et al., 2016[34]	2
Yen et al., 2011[35]	2

(1) Studies with subjects under 18 years old ($n = 2$);
(2) Study with healthy people or studies with a sample composed of individuals without a diagnosis of vestibular dysfunction, or where this diagnosis was not confirmed by specific examination, or that a validated questionnaire was not used to assess vestibular function, or with patients with any medication associated with therapy ($n = 19$);
(3) Studies with only a single session of any type of vestibular rehabilitation not virtual or without virtual reality ($n = 8$);
(4) Patients that taking any medication ($n = 4$);
(5) Cross-sectional or retrospective studies, or studies did not present the results or has no numerical parameters for the meta-analysis ($n = 2$).

 © 2026. The Author(s).

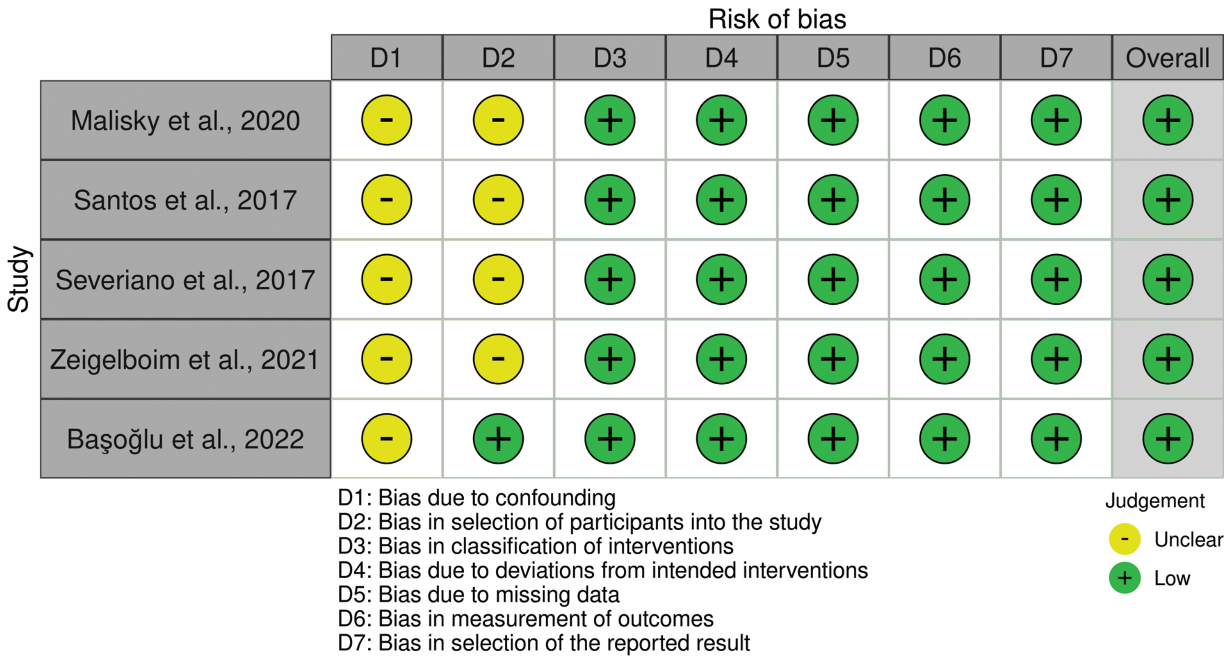

Supplementary Fig 1

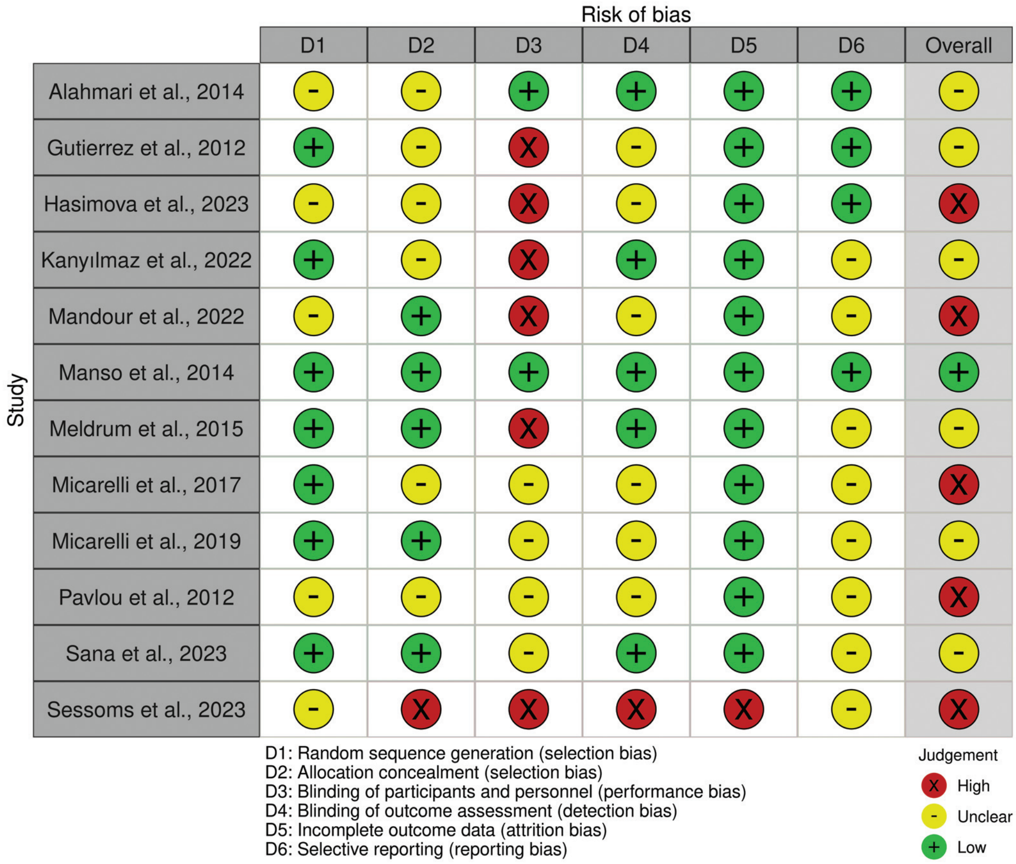

Supplementary Fig 2

 © 2026. The Author(s).

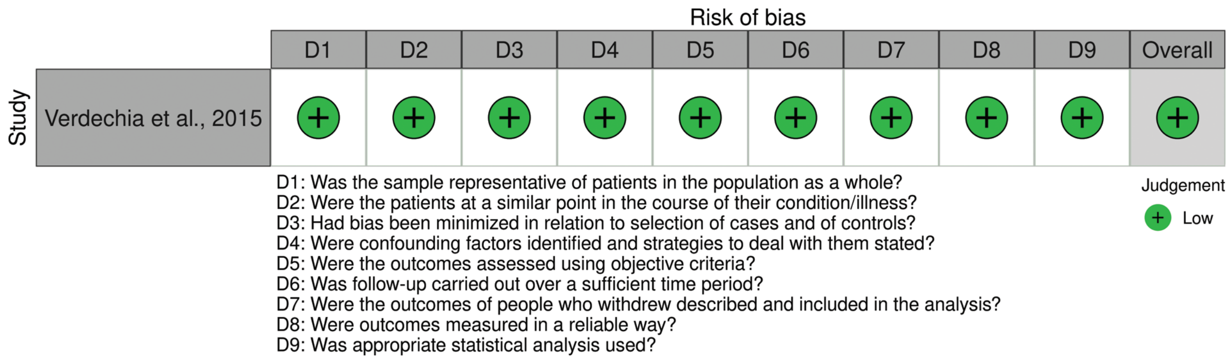

Supplementary Fig 3

Appendix References

1 Alves MLM, Mesquita BS, Morais WS, Leal JC, Satler CE, Dos Santos Mendes FA. Nintendo Wii™ Versus Xbox Kinect™ for Assisting People With Parkinson's Disease. Percept Mot Skills 2018;125 (03):546–565

2 Ansai JH, Aurichio TR, Gonçalves R, Rebelatto JR. Effects of two physical exercise protocols on physical performance related to falls in the oldest old: A randomized controlled trial. Geriatr Gerontol Int 2016;16(04):492–499

3 Bayat A, Pourbakht A, Saki N, Zainun Z, Nikakhlagh S, Mirmomeni G. Vestibular rehabilitation outcomes in the elderly with chronic vestibular dysfunction. Iran Red Crescent Med J 2012;14(11): 705–708

4 Bayat A, Saki N. Effects of Vestibular Rehabilitation Interventions in the Elderly with Chronic Unilateral Vestibular Hypofunction. Iran J Otorhinolaryngol 2017;29(93):183–188

5 Bittar RSMPM, Formigoni LG. Reabilitação vestibular: Uma arma poderosa no auxilio a pacientes portadores de tontura. Braz J Otorhinolaryngol 1999;65(03):266–269

6 Bittar RSMSL, Pedalini MEB, Bottino MA. Repercussão das medidas de correção das comorbidades no resultado da reabilitação vestibular de idosos. Rev Bras Otorrinolaringol 2007;73(03): 295–298

7 Bittar RS, Barros CdeG. Vestibular rehabilitation with biofeedback in patients with central imbalance. Braz J Otorhinolaryngol 2011; 77(03):356–361

8 Black FO, Angel CR, Pesznecker SC, Gianna C. Outcome analysis of individualized vestibular rehabilitation protocols. Am J Otol 2000; 21(04):543–551

9 Ferraz DD, Trippo KV, Duarte GP, Neto MG, Bernardes Santos KO, Filho JO. The Effects of Functional Training, Bicycle Exercise, and Exergaming on Walking Capacity of Elderly Patients With Parkinson Disease: A Pilot Randomized Controlled Single-blinded Trial. Arch Phys Med Rehabil 2018;99(05):826–833

10 Gandolfi M, Geroin C, Dimitrova E, et al. Virtual Reality Telerehabilitation for Postural Instability in Parkinson's Disease: A Multicenter, Single-Blind, Randomized, Controlled Trial. BioMed Res Int 2017;2017:7962826

11 Garcia AP, Ganança MM, Cusin FS, Tomaz A, Ganança FF, Caovilla HH. Vestibular rehabilitation with virtual reality in Ménière's disease. Braz J Otorhinolaryngol 2013;79(03):366–374

12 Kim JH, Jang SH, Kim CS, Jung JH, You JH. Use of virtual reality to enhance balance and ambulation in chronic stroke: a double-blind, randomized controlled study. Am J Phys Med Rehabil 2009; 88(09):693–701

13 Lee NY, Lee DK, Song HS. Effect of virtual reality dance exercise on the balance, activities of daily living, and depressive disorder status of Parkinson's disease patients. J Phys Ther Sci 2015;27 (01):145–147

14 Liao YY, Yang YR, Cheng SJ, Wu YR, Fuh JL, Wang RY. Virtual Reality-Based Training to Improve Obstacle-Crossing Performance and Dynamic Balance in Patients With Parkinson's Disease. Neurorehabil Neural Repair 2015;29(07):658–667

15 Lin Zhi-Cheng CA-Z, Jiang Y-J, Chen D-Z, Ye X-Q, You Y-M. Effects of Virtual Reality Balance Game on Balance Function for Parkinson's Disease. Chin J Rehabil Theory Pract 2016;9:1059–1063

16 Marioni G, Fermo S, Zanon D, Broi N, Staffieri A. Early rehabilitation for unilateral peripheral vestibular disorders: a prospective, randomized investigation using computerized posturography. Eur Arch Otorhinolaryngol 2013;270(02):425–435

17 Marioni G, Fermo S, Lionello M, et al. Vestibular rehabilitation in elderly patients with central vestibular dysfunction: a prospective, randomized pilot study. Age (Dordr) 2013;35(06):2315–2327

18 Meldrum D, Herdman S, Moloney R, et al. Effectiveness of conventional versus virtual reality based vestibular rehabilitation in the treatment of dizziness, gait and balance impairment in adults with unilateral peripheral vestibular loss: a randomised controlled trial. BMC Ear Nose Throat Disord 2012;12:3

19 McConville KMV, Virk S. Evaluation of an electronic video game for improvement of balance. Virtual Real (Walth Cross) 2012;16 (04):315–323

20 Morozetti PG, Ganança CF, Chiari BM. Comparação de diferentes protocolos de reabilitação vestibular em pacientes com disfunções vestibulares periféricas. J Soc Bras Fonoaudiol 2011; 23:44–50

21 Nishiike S, Okazaki S, Watanabe H, et al. The effect of visual-vestibulosomatosensory conflict induced by virtual reality on postural stability in humans. J Med Invest 2013;60(3-4):236–239

22 Özgönenel L, Çağırıcı S, Çabalar M, Durmuşoğlu G. Use of Game Console for Rehabilitation of Parkinson's Disease. Balkan Med J 2016;33(04):396–400

23 Park MJ, Kim DJ, Lee U, Na EJ, Jeon HJ. A Literature Overview of Virtual Reality (VR) in Treatment of Psychiatric Disorders: Recent Advances and Limitations. Front Psychiatry 2019;10:505

24 Pedreira G, Prazeres A, Cruz D, et al. Virtual games and quality of life in Parkinson's disease: A randomised controlled trial. Adv Parkinsons Dis 2013;02(04):97–101

25 Pompeu JE, Mendes FA, Silva KG, et al. Effect of Nintendo Wii™-based motor and cognitive training on activities of daily living in patients with Parkinson's disease: a randomised clinical trial. Physiotherapy 2012;98(03):196–204

26 Purvis CK, Jones M, Bailey JO, Bailenson J, Taylor CB. Developing a Novel Measure of Body Satisfaction Using Virtual Reality. PLoS One 2015;10(10):e0140158

27 Ribas CG, Alves da Silva L, Corrêa MR, Teive HG, Valderramas S. Effectiveness of exergaming in improving functional balance, fatigue and quality of life in Parkinson's disease: A pilot randomized controlled trial. Parkinsonism Relat Disord 2017;38:13–18

 © 2026. The Author(s).

28 Shen X, Mak MK. Balance and Gait Training With Augmented Feedback Improves Balance Confidence in People With Parkinson's Disease: A Randomized Controlled Trial. Neurorehabil Neural Repair 2014;28(06):524–535
29 Shih MC, Wang RY, Cheng SJ, Yang YR. Effects of a balance-based exergaming intervention using the Kinect sensor on posture stability in individuals with Parkinson's disease: a single-blinded randomized controlled trial. J Neuroeng Rehabil 2016;13(01):78
30 van den Heuvel MR, Kwakkel G, Beek PJ, Berendse HW, Daffertshofer A, van Wegen EE. Effects of augmented visual feedback during balance training in Parkinson's disease: a pilot randomized clinical trial. Parkinsonism Relat Disord 2014;20(12):1352–1358
31 Salisbury C, Man MS, Bower P, et al. Management of multimorbidity using a patient-centred care model: a pragmatic cluster-randomised trial of the 3D approach. Lancet 2018;392 (10141):41–50
32 Sparrer I, Duong Dinh TA, Ilgner J, Westhofen M. Vestibular rehabilitation using the Nintendo® Wii Balance Board – a user-friendly alternative for central nervous compensation. Acta Otolaryngol 2013;133(03):239–245
33 Suárez H, Suárez A, Lavinsky L. Postural adaptation in elderly patients with instability and risk of falling after balance training using a virtual-reality system. Int Tinnitus J 2006;12(01):41–44
34 Yang WC, Wang HK, Wu RM, Lo CS, Lin KH. Home-based virtual reality balance training and conventional balance training in Parkinson's disease: A randomized controlled trial. J Formos Med Assoc 2016;115(09):734–743
35 Yen CY, Lin KH, Hu MH, Wu RM, Lu TW, Lin CH. Effects of virtual reality-augmented balance training on sensory organization and attentional demand for postural control in people with Parkinson disease: a randomized controlled trial. Phys Ther 2011;91(06): 862–874

 © 2026. The Author(s).
